# High Efficient Solar Cell Based on Heterostructure Constructed by Graphene and GaAs Quantum Wells

**DOI:** 10.1002/advs.202204058

**Published:** 2022-11-17

**Authors:** Xutao Yu, Yue Dai, Yanghua Lu, Chang Liu, Yanfei Yan, Runjiang Shen, Zunshan Yang, Lixuan Feng, Lijie Sun, Yong Liu, Shisheng Lin

**Affiliations:** ^1^ College of Information Science and Electronic Engineering Zhejiang University Hangzhou 310027 P. R. China; ^2^ State Key Laboratory of Space Power Technology Shanghai Institute of Space Power Sources Shanghai 200245 P. R. China; ^3^ State Key Laboratory of Modern Optical Instrumentation Zhejiang University Hangzhou 310027 P. R. China

**Keywords:** carrier multiplication, graphene, heterostructure, hot carriers, quantum wells

## Abstract

Despite the fascinating optoelectronic properties of graphene, the power conversion efficiency (PCE) of graphene based solar cells remains to be lifted up. Herein, it is experimentally shown that the graphene/quantum wells/GaAs heterostructure solar cell can reach a PCE of 20.2% and an open‐circuit voltage (*V*_oc_) as high as 1.16 V at 90 K. The high efficiency is a result of carrier multiplication (CM) effect of graphene in the graphene/GaAs heterostructure. Especially, the external quantum efficiency (EQE) in the ultraviolet wavelength can be improved up to 72.2% based on the heterostructure constructed by graphene/In_0.15_Ga_0.85_As/GaAs_0.75_P_0.25_ quantum wells/GaAs. The EQE increases as the light wavelength decreases, which indicates more carriers can be effectively excited by the higher energy photons through CM effect. Owing to these physical characters, the graphene/GaAs heterostructure solar cell will provide a possible way to exceed Shockley–Queisser (S–Q) limit.

## Graphical abstract

Graphene/QWs/GaAs heterostructure solar cell shows a higher EQE of 72.2% in the ultraviolet wavelength than that in the whole visible light range, as an experimental evidence of effective use of the carrier multiplication effect of graphene. This work has transferred the signal that graphene/semiconductor heterostructure‐based solar cell can be very competitive for overcoming the S–Q limit.

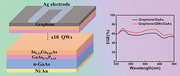

## 1 Introduction

Graphene is composed of sp^2^ hybrid carbon atoms with the honeycomb lattice structure.^[^ ^1^ ^]^ As a member of a large family of two‐dimensional (2D) layered materials, graphene has many outstanding electrical, optical and mechanical properties, and widely applied in transistors,^[^ ^2^ ^]^ optoelectronic^[^ ^3^ ^]^ and wearable electronic devices.^[^ ^4^ ^]^ Also, numerous efforts have been devoted to realize graphene based high efficiency solar cells, applied in graphene electrodes,^[^ ^5^ ^]^ graphene electron transport layers,^[^ ^6^ ^]^ graphene/semiconductor heterostructure,^[^ ^7^ ^]^ and so on. The power conversion efficiency (PCE) of 18.5% has been realized with a designed graphene–dielectric–graphene gating structure and highly tunable Fermi level of graphene in our previous work.^[^ ^8^ ^]^ But, in order to overcome Shockley–Queisser (S–Q) limit, inhibiting the intrinsic thermalization energy loss channel is critical for improving the PCE of solar cell.^[^ ^9^ ^]^ In this context, how to make efficient use of hot carriers (HCs) and decrease the main energy losses become a particularly critical problem.^[^ ^9^, ^10^ ^]^ Despite the existence of generating more carriers through interband carrier–carrier scattering in the conventional bulk materials, HCs can hardly be extracted because of the rapid relaxation by carrier–phonon scattering before arriving to the band edges. However, in graphene, owing to an atomic thin crystal with electrons described by Dirac equations, which points to its linear band gap structure near the Dirac point, and are predicted to illustrate highly efficient carrier multiplication (CM) effect to utilize HCs more efficient. Furthermore, graphene has been theoretically predicted and experimentally observed that HCs can be exploited to realize a significant CM through the Auger process, as a feasible path to decrease the thermal energy loss.^[^ ^11^ ^]^ In addition, in spite of the presence of directly competing process of Auger recombination and phonon‐induced recombination, impact ionization is efficient enough to realize a prominent CM in graphene.^[^ ^12^ ^]^

Due to the weak light‐absorbing ability of graphene, the selection of related semiconductor materials to combine with it is also particularly important. Conventional bulk materials, such as GaAs, which has a suitable bandgap of 1.42 eV to harvest the maximum possible solar spectrum, sufficiently high carrier mobility and distinctive phononic bandgap, formatting a hot‐phonon bottleneck to limit the excess energy redistribution into the lattice, which can be a suitable HCs absorber.^[^ ^13^ ^]^ The externally injected photogenerated carriers from graphene can effectively enhance the hot‐phonon bottleneck effect of GaAs, which will lead to a prolonged HCs cooling process and be beneficial to improve the PCE of solar cell.

In this work, we reveal an efficient CM effect of graphene, and have demonstrated graphene/GaAs heterostructure solar cell with an external quantum efficiency (EQE) of 67.8% in ultraviolet wavelength, which is higher than that in the whole visible wavelength. Especially at low temperature, graphene/GaAs heterostructure solar cell can excite and utilize HCs more effectively. Furthermore, through integration of GaAs_0.75_P_0.25_ and In_0.15_Ga_0.85_As multi quantum wells (QWs), we have demonstrated the graphene/QWs/GaAs heterostructure solar cell with the *V*_oc_ and PCE of 1.16 V and 20.2% at the temperature of 90 K, respectively. Notably, at 300 K, the EQE of graphene/QWs/GaAs heterostructure has reached 72.2% at ultraviolet wavelength. The higher EQE indicates that HCs in graphene can be exploited to realize a more significant CM through the integration of QWs. This research not only points out that graphene/GaAs heterostructure solar cell could provide the feasibility of harvesting HCs effectively to restrict thermalization loss channels, and further to overcome the S–Q limit, but also demonstrates its potential applications as energy source in extremely low temperature environments.

## 2 Results and Discussion

**Figure 1:**
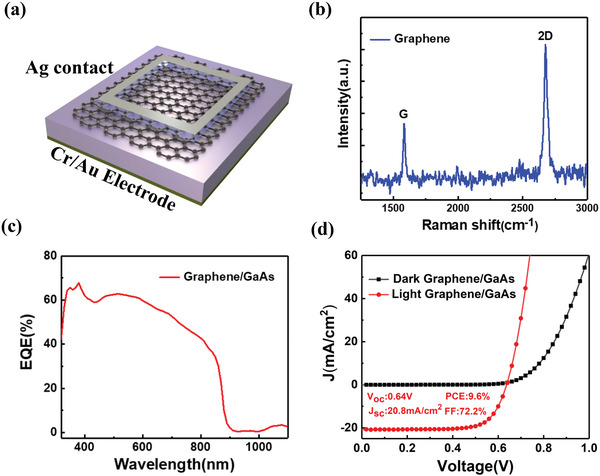
Device structure and characteristic test of graphene/GaAs heterostructure solar cell. a) Schematics structure of the graphene/GaAs heterostructure solar cell. b) Raman characterization of graphene onto the SiO_2_/Si substrate after wet transferring. c) EQE of solar cell with the scanning wavelength from 320 nm to 1100 nm. d) *J*–*V* characteristic curve: black and red curves represent dark condition and exposed in AM 1.5 light source, individually. The active area of graphene/GaAs heterostructure solar cell was 0.03 cm^2^.

**Figure** **1**a illustrates the three‐dimensional (3D) architecture of graphene/GaAs heterostructure solar cell. The preparation of graphene and GaAs substrate have been mentioned in the “Experimental Section.” As the thinnest 2D material, the thickness of a single layer of graphene is only 0.34 nm,^[^ ^14^ ^]^ which is conducive to the formation of ultra‐shallow heterojunction on the material surface. Simultaneously, the formation of the ultra‐high built‐in electric field between the interfaces is also beneficial to lead a faster carriers separation process. In Figure 1b, the Raman spectroscopy characterization of used graphene shows the characteristic peaks locate at 1588.5 and 2676.2 cm^−1^, corresponding to G and 2D peaks, individually. Around the D peak (about 1350 cm^−1^), the intensity is pretty weak, which indicates few defects in graphene. Compared with 1580 cm^−1^ of pure graphene, as‐grown graphene is p‐type doped after the wet transferring process. The intensity of 2D peak is higher than G peak and the shape is single peak, which reflect that as‐grown graphene is a monolayer.^[^ ^15^ ^]^

As shown in Figure 1c, EQE test started from 320 nm and ended near 1100 nm. It is worth noting that the EQE has reached the maximum value 67.8% at ultraviolet wavelength, which is also obviously higher than that at the whole visible wavelength. According to the intrinsic absorption of semiconductor theory, wavelength of light that can be absorbed by the semiconductor is calculated as follows:

(1) $$\lambda \leq \frac{h\cdot c}{E_{g}}$$

where *λ* is the wavelength of absorbed light, *h* is Planck's constant, *c* is the speed of light, *E*_g_ is the band gap of semiconductor material. According to Equation (1), the photon energy of short‐wavelength is several times than the band gap of GaAs material (the band gap of GaAs is about 1.42 eV). Therefore, the more carriers can be photoexcited as high energy carriers owing to absorption characteristics in short‐wavelength even though they are beyond the band edges of substrate materials, as the original definition of HCs. Then, HCs will have an ultra‐fast relaxation and lead to a significant CM, which are converted into a part of the photocurrent as result shown in the EQE curve. So, the value of EQE points that HCs have been utilized effectively, which also represents the feasibility of graphene/GaAs heterostructure to reduce thermal energy loss to further to enhance the PCE of solar cell.^[^ ^9^ ^]^ Meanwhile, the *J*–*V* curve of graphene/GaAs heterostructure solar cell has been measured. As shown in Figure 1d, the *J*–*V* curves exhibit superior photoelectric response based on the graphene/GaAs heterostructure at room temperature under AM 1.5, where the *V*_oc_, *J*_sc_, and PCE are 0.64 V, 20.8 mA cm^−2^, and 9.6%, respectively. Although the basic efficiency of graphene/GaAs heterostructure solar cell is not impressive enough, it has demonstrated the effective absorption to generate HCs in short‐wavelength, which will be a critical point to consider how to utilize HCs more effectively for realizing the high efficiency solar cells.

**Figure 2:**
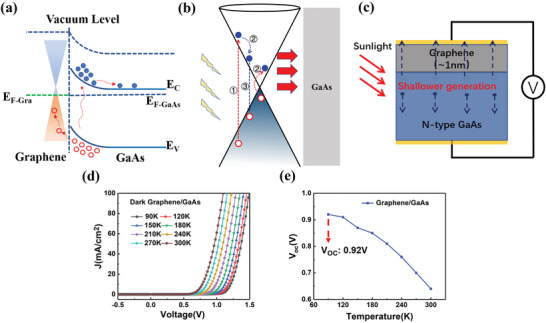
The physical schematic of graphene/GaAs heterostructure solar cell. a) Schematic band structure of graphene/GaAs heterostructure. b) Main kinds of processes of HCs in graphene/GaAs heterostructure: ①light excitation; ②CM; ③ recombination. c) Schematic diagram of carrier kinetics in graphene/GaAs solar cell with the sunlight. d) The dark *J*–*V* curve and e) the *V*_oc_ measurement of graphene/GaAs heterostructure solar cell under the AM 1.5 with ambient temperature varying from 300 to 90 K. The active area of device is 0.03 cm^2^.

The mechanism of graphene/GaAs heterostructure has been shown in **Figure** **2**. Schematic diagram of graphene/GaAs heterostructure has been exhibited in Figure 2a. Due to the wet transfer process, monolayer graphene has been p‐type doped, where has been shown in the location of Fermi level (*E*_F‐Gra_). When the sunlight irradiates on the surface of active area of graphene/GaAs solar cell, the GaAs substrate absorb the photon energy and generates electron–hole pairs. Subsequently, as shown in Figure 2a, due to the built‐in electric field, the electrons and holes will be accelerated and collected on the GaAs back electrode and graphene surface electrode, respectively.

As mentioned above, the graphene/GaAs heterostructure solar cell shows obvious absorption characteristics in ultraviolet wavelengths. In Figure 2b (process ①), photons in the ultraviolet wavelength can excite high energy electron–hole pairs in graphene. Those high energy carriers not in thermal equilibrium with the lattice are regarded as HCs. Subsequently, when the end of optical excitation, an ultrafast relaxation will occur. These carriers will set a new equilibrium among themselves by impact excitation and excite an electron from valance band into conduction band to realize efficient result of carrier–carrier scattering, to decrease their own excess energy (process ②). Besides, comparing with impact ionization, the Auger recombination, as a competing process, where electrons scatter from conduction band into valence band and relax to a lower energy state, the excess energy is transferred to surrounding electron into a higher energy state (process ③). The whole process is competitive to realize CM, which generates multiple electron–hole pairs by the absorption of single photon, leading to convert an efficient photocurrent and a high quantum efficiency especially in ultraviolet wavelength (Figure 1c). Also, due to the presence of hot‐phonon bottleneck effect, leading to a prolonged HCs cooling process in graphene, which creates the extremely favorable conditions to extract and form effective photocurrent. The extra holes generated by the aforementioned process and contribute to the photocurrent. Since monolayer graphene is only atomic‐layer thick, as shown in Figure 2c the heterostructure of graphene/GaAs contact interface, HCs at the contacted interface will be extracted quickly and collected by the surface silver electrode. These excellent properties of graphene enable the graphene/GaAs heterostructure to make use of excess photon energy by separating and transporting HCs before the cooling process despite of picosecond scale of duration, contributing to an efficient photocurrent in the end.

Also, there are many fundamental theoretical calculations to discuss limit factors of CM in graphene, such as applied magnetic field, pump fluence, ambient temperature, and so on.^[^ ^16^ ^]^ Further to explore the feasible strategy to utilize HCs intuitively and sufficiently, the variable temperature experiments with certain gradients have been designed. The dark *J*–*V* curves with different temperature of graphene/GaAs heterostructure solar cell have been measured in Figure 2d, which not only indicate the well interface contact under extremely low temperature but also prove the increase of heterostructure *V*_oc_ synchronously.

Additionally, in Figure 2e, the *V*_oc_ of graphene/GaAs heterostructure solar cell varied with different temperature have been measured in the low temperature optical measurement system. As the temperature gradually decreasing, the *V*_oc_ has increased to 0.92 V at the 90 K. Contrasted with the *V*_oc_ at the 300 K, the *V*_oc_ has been increased 43.8% at the 90 K. In graphene, due to the gradual decrease of ambient temperature to improve carrier mobility,^[^ ^17^ ^]^ speed up the cooling of the internal phonon transmission channel, and lead to the lower electron–photon coupling, which will delay cooling process of HCs. Simultaneously, a lower temperature will result in a high‐efficient impact excitation to decrease an initial occupation and promote the CM effect.^[^ ^16c^ ^]^ Eventually, the decreased temperature would lead to a performance enhancement of the graphene/GaAs heterostructure solar cell. Also, the enhanced PCE of solar cell (Figure S1, Supporting Information) has showed that low temperature is beneficial to the absorption of HCs for graphene/GaAs heterostructure. However, it is worth considering that the *J*_sc_ of graphene/GaAs heterostructure decreases as the temperature decreased (Figure S2, Supporting Information). Surely, though the inter‐band carrier–carrier scattering leading to CM due to HCs, how to decrease carrier recombination at the interface is a key point to further improve conversion efficiency effectively.

**Figure 3:**
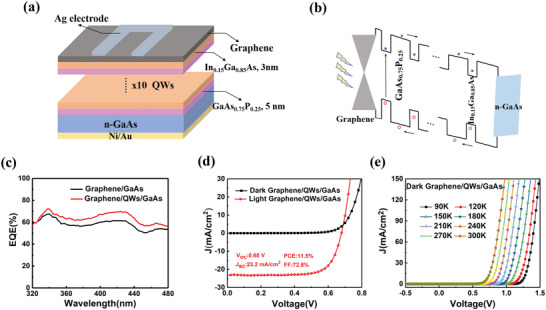
Schematic and performance characterization of graphene/QWs/GaAs solar cell. a) Schematic representation graphene/QWs/GaAs solar cell: the QWs are composed of GaAs_0.75_P_0.25_ /In_0.15_Ga_0.85_As with 10 periods. b) Schematic energy band diagram of graphene/QWs/GaAs structure. c) The comparation of EQE test during the short‐wavelength from 320 to 480 nm: black and red curves represent graphene/GaAs and graphene/QWs/GaAs structure, individually. d) *J*–*V* curve of graphene/QWs/GaAs solar cell at the room temperature: black and red curves represent dark condition and under AM 1.5, individually. e) *J*–*V* characteristics curves under the dark state with various temperature from 300 to 90 K. The active area of graphene/QWs/GaAs heterostructure solar cell is 0.03 cm^2^.

In addition, quantum wells (QWs), have been studied in multi‐junction solar cells, as an effective structure to optimize the absorption of solar spectra and reduce the thermalization losses of solar cells, which ultimately lead to increase the number of photogenerated carriers to improve the performance of solar cells.^[^ ^18^ ^]^ Additionally, although the introduced QWs into conventional bulk solar cells have made a breakthrough either in multi‐junction or single junction solar cells, while considering the material lattice matching, the effective utilization of HCs is tough to achieve. Due to the fatal flaw of conventional bulk solar cells, the width of the space charge region is so large that the HCs cannot be extracted in time, resulting in the recombination of carriers and limiting the further improvement of solar cells efficiency. So, optimizing the structure is critical point to the further investigation to increase the efficiency of solar cells. Herein, we have demonstrated the structure of graphene/QWs/GaAs solar cell. In **Figure** **3**a, the schematic structure of graphene/QWs/GaAs solar cell has been showed. There are fewer dislocations by adjusting the alloy compositions, the thickness of wells and barriers. Based on the universal stress–balance condition, GaAsP and InGaAs are grown epitaxially with the similar lattice constant as the GaAs substrate, which is effective to decrease the stress or even zero stress among the different layers. So, the alloy layers are designed as 5 nm GaAs_0.75_P_0.25_ and 3 nm In_0.15_Ga_0.85_As, forming a combination of 10 periods QWs. Subsequently, monolayer graphene is transferred onto the In_0.15_Ga_0.85_As QWs and the Ag paste is covered on the graphene as the surface electrode.

The photogenerated carriers transport process has been shown in Figure 3b. When the graphene and GaAs substrate absorb incident photon, a part of photon will also be absorbed by the periodic wells. The band gap of GaAs_0.75_P_0.25_ and In_0.15_Ga_0.85_As are 1.63 and 1.21 eV, individually. Due to the difference of bandgap between GaAs and QWs materials, the absorption spectrum can be expanded effectively, where the QWs epitaxially grown on GaAs are undoped. So, the light at the edge of the spectrum can be utilized by the QWs as the intrinsic region, which also absorb the energy of photon and generate extra electron–hole pairs. Simultaneously, thin enough depletion layer (Note S1, Supporting Information) and ultra‐high built‐in electric field of graphene/QWs/GaAs heterostructure, can be more efficient to ensure sperate the photogenerated electron–hole pairs and transport the carriers in time to decrease the surface recombination loss. The total thickness of QWs is only 80 nm, which also ensures that HCs have a fast transport process to be extracted near the electrode. In Figure 3b, as the main two part of photocurrent, HCs generated at the interface of graphene and photogenerated carriers in the QWs as intrinsic region, they will escape from the potential well or tunnel through the barriers under the built‐in electric field, coming into final current in the end.^[^ ^19^ ^]^ Certainly, the figure of carriers’ transportation is the mainly embodied process, the extended absorption to generate electron–hole pairs and recombination of part photogenerated carriers are also existing in the QWs. In order to compare the effect by integrating QWs structure, the EQE test especially during the short‐wavelength (320–480 nm) have been measured in Figure 3c. The red curve, which represents the graphene/QWs/GaAs structure, demonstrates higher quantum efficiency (the maximum value is 72.2% at 340 nm) than black curve over the whole short‐wavelength. So, there are more HCs to be utilized and collected at the electrode to increase the performance of graphene/GaAs heterostructure solar cells.

Furthermore, the *J*–*V* characteristic curve have been measured at room temperature in Figure 3d. Inside *J*–*V* curve at the dark condition, the extremely small leakage indicates that the solar cell has excellent junction characteristics. Compared with the graphene/GaAs heterostructure, both the *J*_sc_ and *V*_oc_ of the graphene/QWs/GaAs heterostructure have a certain improvement under the sunlight. Especially, the *J*_sc_ has reached to 23.2 mA cm^−2^, which has promoted 11.5% compared with graphene/GaAs heterostructure (the *J*_sc_ is 20.8 mA cm^−2^). Owing to the multi QWs structure, the extra absorbed photons to generate photogenerated carriers, which contribute to the increased photocurrent. Hence, the PCE of solar cell based on the graphene/QWs/GaAs structure has been greatly increased to 11.5%. In the same way, the variable temperature experiments have been also measured. As the experimental data shown in Figure 3e, the dark state *J*–*V* curves have illustrated that graphene/QWs/GaAs heterostructure solar cell still exhibit well stability in the low temperature environment. Additionally, the variation of potential difference is rather apparent, when the temperature of environment gradually decreases. Simultaneously, comparing with the *J*–*V* curve of graphene/GaAs heterostructure solar cell (Figure 2d), due to the QWs structure as the intrinsic region, the potential difference shown in the curve has also been increased.

**Figure 4:**
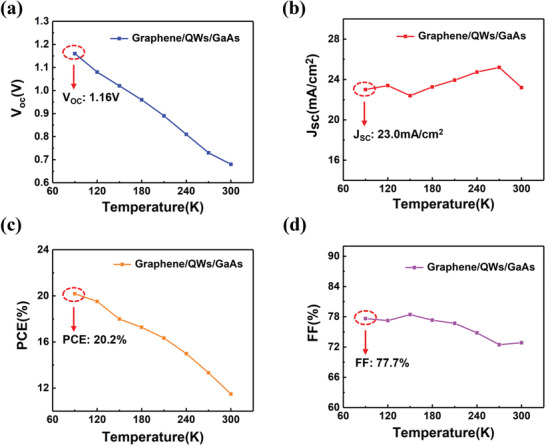
The performance characterization test of graphene/QWs/GaAs heterostructure solar cell. a) The *V*_oc_, b) *J*_sc_, c) PCE, and d) FF of graphene/QWs/GaAs heterostructure solar cell under the temperature of 300, 270, 240, 210, 180, 150, 120, 90 K. All the measurement is under AM 1.5 and the active area of graphene/QWs/GaAs heterostructure solar cells are 0.03 cm^2^.

In **Figure** **4**, the main parameters *J*_sc_, *V*_oc_, PCE, and FF of graphene/QWs/GaAs heterostructure solar cell have been measured with various temperature. As shown in Figure 4a, the *V*_oc_ gradually increases from 0.68 to 1.16 V as the temperature decreasing from 300 to 90 K. Compared with the *V*_oc_ of graphene/GaAs heterostructure solar cell (Figure 2e), the *V*_oc_ has been increased over 26% at the temperature of 90 K. However, as for conventional PN GaAs solar cell, related discussions have been shown in Figure S3 and Note S2 (Supporting Information), which can be explained for the change of band gap at various temperature. And for graphene/QWs/GaAs heterostructure, QWs structure can offer a continuous carrier density of states and leads to a broadband solar energy absorption, further to opens up a phononic bandgap, resulting in the slow cooling of HCs.^[^ ^20^ ^]^ In addition, due to the increase of the HCs relaxation time at low ambient temperature, the number of HCs that can be efficiently collected will increase, causing the dynamic splitting of the quasi‐Fermi level and resulting in an enhanced open‐circuit voltage.

Figure 4b shows that the *J*_sc_ of graphene/QWs/GaAs heterostructure vary with the temperature. Although the ambient temperature decreases, it is worth noting that the value of *J*_sc_ are almost unchanged. Generally, the current of PN junction solar cell are described as:

(2) $$I\;=I_{L}\;-I_{F}\;$$

where *I*_L_ is the photogenerated current, *I*_F_ is the forward current caused by photogenerated voltage. And the photogenerated current *I*_L_ can be expressed by:

(3) $$I_{L}=\;eA\left(L_{p}+L_{n}+W\right)g^{\prime }$$

where *e* is the electron charge, *A* is the area of the PN junction, *L*_p_ and *L*_n_ are the diffusion length of minority carrier, *W* is the depletion layer width and *g*′ is the generation rate of electron–hole pairs. According to Equations (2) and (3),^[^ ^21^ ^]^ when the temperature decreases, the diffusion length decreases, leading to the loss of *J*_sc_. In a conventional solar cell based on PN junction of bulk material, photogenerated carriers need to travel through emitter region with thickness over 100 nm before being collected by the front contact. Thus, the decrease of temperature has an obvious effect on *J*_sc_ of the conventional solar cell. As for graphene/QWs/GaAs heterostructure solar cell, the QWs structure can absorb additional photons to generate photogenerated carriers effectively and limit the carrier recombination loss during the transport process, which is beneficial to increase the number of carriers. Additionally, the carrier mobility in graphene will be increased in a lower temperature,^[^ ^17^ ^]^ and ensure the faster collection of carriers in graphene, which can reduce the influence of the diffusion length change caused by the temperature drop on the *J*_sc_. Due to those excellent characteristics of graphene/QWs/GaAs heterostructure, the PCE can improve considerably. As shown in Figure 4c, the highest PCE of 20.2% has been achieved at 90 K, which demonstrates the possibility of application as an energy source in extremely low temperature environments. Meanwhile, the FF has also been calculated in Figure 4d. As the temperature decreasing, the FF has increased gradually from 72.8% to 78% and stable in 77.7% at 90 K. As mentioned above, all excellent characteristics of graphene/QWs/GaAs heterostructure solar cell, which points out a feasible way to utilize and collect HCs under low temperature by prolonging the cooling process and realizing a significant CM effect, and enhance the performance of solar cell based on graphene/GaAs heterostructure in the end.

## 3 Conclusion

In this work, we have demonstrated graphene/GaAs heterostructure solar cell, owing to the excellent characteristics of graphene to realize a significant CM through absorbing a high energy photon. Especially in the low temperature environment, effectively delay the cooling process of HCs for transmission and collection, which leads to enhance performance of graphene/GaAs heterostructure solar cell. Further to introduce the GaAs_0.75_P_0.25_ and In_0.15_Ga_0.85_As multi QWs structure, the graphene/QWs/GaAs heterostructure solar cell has been demonstrated, where the maximum value 72.2% of EQE has been achieved at ultraviolet wavelength. By enhancing the absorption of the edge of spectrum and minimizing HCs cooling during extraction, which has achieved the PCE of 11.5% at the room temperature. When the ambient temperature decreases, the graphene/QWs/ GaAs heterostructure solar cell also shows the excellent stability and performance, the *V*_oc_ and PCE have been achieved to 1.16 V and 20.2% individually at the 90 K. As the representative of 2D van der Waals heterostructure, graphene/GaAs heterostructure plays a crucial role in high efficiency solar cell, which is promising to be the next generation photovoltaic technology to beyond the S–Q limit by means of further optimizing the structure and reducing the extrinsic loss channels to utilize the HCs more sufficiently.

## 4 Experimental Section

### The Preparation of Graphene

Graphene was grown on a cooper foil with chemical vapor deposition (CVD) technique using CH_4_ and H_2_ at the ratio of 5:1 as the reaction gas sources at 1000 °C for 30 min. Polymethyl methacrylate (PMMA) was spin‐coated on the front surface of cooper to be a supporting and protective layer. Then, hydrochloric acid and CuSO_4_ mixed solution was prepared to etch the copper and obtained monolayer graphene for about 2 h. After that, the graphene was transferred to deionized water to clean up.

### Fabrication of Graphene/GaAs Solar Cell

The *N*‐type GaAs substrate was doped with silicon at a density of *N*_d_ = 2 × 10^17^ cm^−3^. 10 nm Ti was thermally evaporated on back surface of GaAs to form an ohmic contact, followed by a 50 nm Au rear electrode. Then, graphene was transferred onto GaAs substrate and the PMMA of the surface of graphene was removed later by acetone, isopropanol and deionized water in turn for 15 min, individually. Silver paste was pasted onto graphene as a front contact electrode and annealed at 105 °C for about 15 min on the hot plate.

### Fabrication of Graphene/QWs/GaAs Solar Cell

The *N*‐type GaAs substrate was doped with silicon at a density of *N*_d_ = 2 × 10^18^ cm^−3^. QWs are epitaxial growth on the GaAs substrate with alloy layers are designed as 5 nm GaAs_0.75_P_0.25_ and 3 nm In_0.15_Ga_0.85_As layers, forming a combination of 10 periods. 10 nm Ti was thermally evaporated on back surface of GaAs to form an ohmic contact, followed by a 50 nm Au rear electrode. Then, graphene was transferred onto In_0.15_Ga_0.85_As layers and the PMMA of the surface of graphene was removed later by acetone, isopropanol and deionized water in turn for 15 min, individually. Silver paste was pasted onto graphene as a front contact electrode and annealed at 105 °C for about 15 min on the hot plate.

### Fabrication of Single‐Junction GaAs Solar Cell

A conventional single‐junction GaAs solar cell with N on P structure consisting of a 100 nm *N*‐type GaAs layer with the doping density of *N*_d_ = 2 × 10^18^ cm^−3^ and a 3 µm P‐type GaAs layer of *N*_a_ = 1 × 10^17^ cm^−3^ was used as a control group. The rear contact of the PN GaAs solar cell was made of Au–Ge–Ni alloy, and the front electrode was made into grid structure with Au.

### Characterization

The *I*–*V* characteristics were measured under the standard spectrum of AM 1.5 at temperatures ranging from 90 to 300 K with a step length of 30 K and an error of ± 0.2 K in the low temperature optical measurement system. The effective working area of the solar cells were measured and calculated with Image Pro software. EQE tests were measured by Enlitech QE‐R system. After transferring to SiO_2_/Si substrate (oxide layer thickness was 270 nm), monolayer graphene was characterized by Raman spectroscopy (Renishaw inVia Reflex) with the excitation wavelength of 532 nm and the sampling interval of 1 nm.

## Conflict of Interest

The authors declare no conflict of interest.

## Author Contributions

S.L. designed the experiments, discussed the results, and conceived the study. X.Y. carried out the experiments, discussed the results, and wrote the paper. Y.D., Y.L., C.L., Y.Y., R.S., Z.Y., L.F., L.S., and Y.L. discussed the results and assisted with experiments. All authors contributed to the preparation of the manuscript.

## Supporting information

Supporting Information

## Data Availability

The data that support the findings of this study are available from the corresponding author upon reasonable request.
