# Supplementary material for: High Efficient Solar Cell Based on Heterostructure Constructed by Graphene and GaAs Quantum Wells
Source: Adv Sci (Weinh). 2022 Nov 17;10(2):2204058. doi: 10.1002/advs.202204058 (PMC9839879; doi:10.1002/advs.202204058)
Supplement: Supplementary file 1 — Supporting Information [file ADVS-10-2204058-s001.pdf]

ADVANCED SCIENCE
Open Access

Supporting Information

for *Adv. Sci.*, DOI 10.1002/advs.202204058

High Efficient Solar Cell Based on Heterostructure Constructed by Graphene and GaAs Quantum Wells

*Xutao Yu, Yue Dai, Yanghua Lu, Chang Liu, Yanfei Yan, Runjiang Shen, Zunshan Yang, Lixuan Feng, Lijie Sun, Yong Liu and Shisheng Lin**

High efficient solar cell based on heterostructure constructed by graphene and GaAs quantum wells

Xutao Yu[1], Yue Dai [1], Yanghua Lu[1], Chang Liu[1], Yanfei Yan[1], Runjiang Shen[1]

Zunshan Yang[1], Lixuan Feng[1], Lijie Sun[2], Yong Liu[2] and Shisheng Lin[1,3]*

[1]College of Information Science and Electronic Engineering, Zhejiang University, Hangzhou, 310027, P. R. China

[2]State Key Laboratory of space power technology, Shanghai Institute of Space Power Sources, Shanghai, 200245, P. R. China

[3]State Key Laboratory of Modern Optical Instrumentation, Zhejiang University, Hangzhou, 310027, P. R. China

[*]Correspondence: shishenglin@zju.edu.cn.

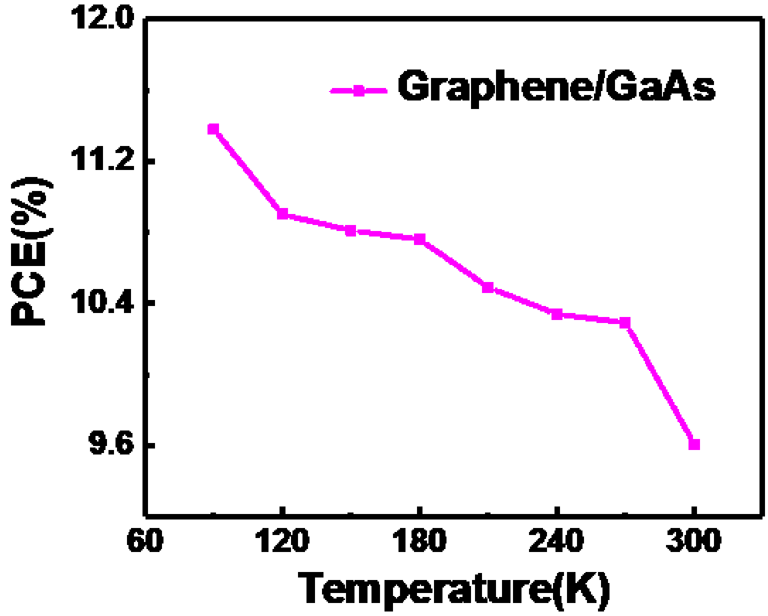

Figure S1. PCE vary with different temperature based on the graphene/GaAs heterostructure solar cell.

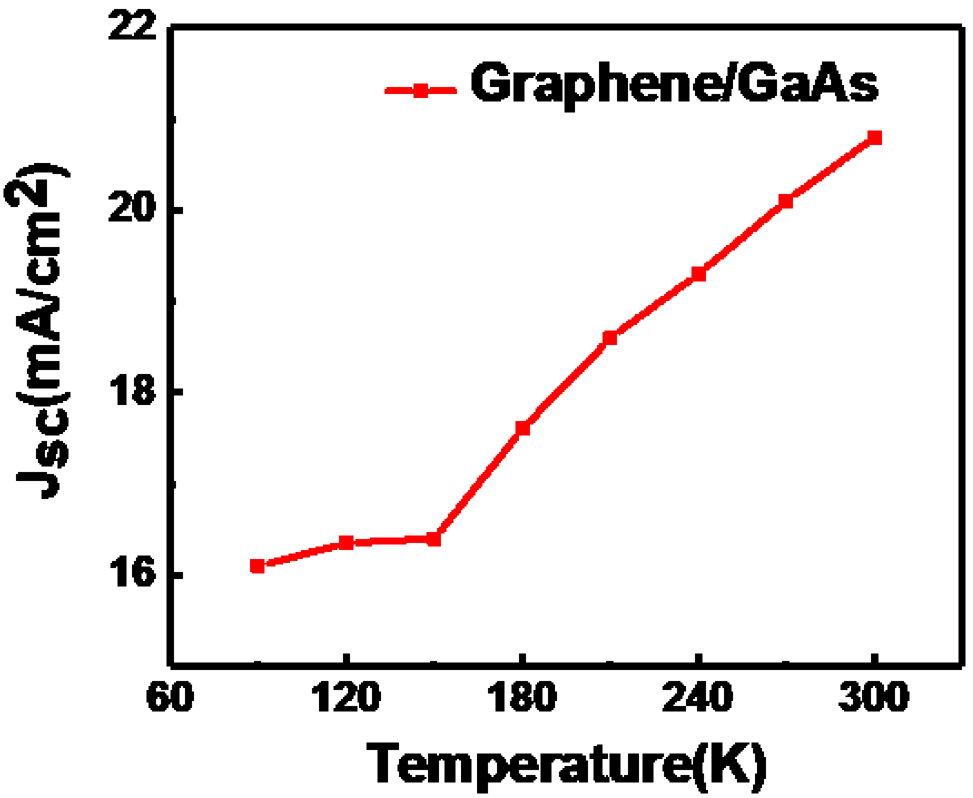

Figure S2. J_{sc} vary with different temperature based on the graphene/GaAs heterostructure solar cell.

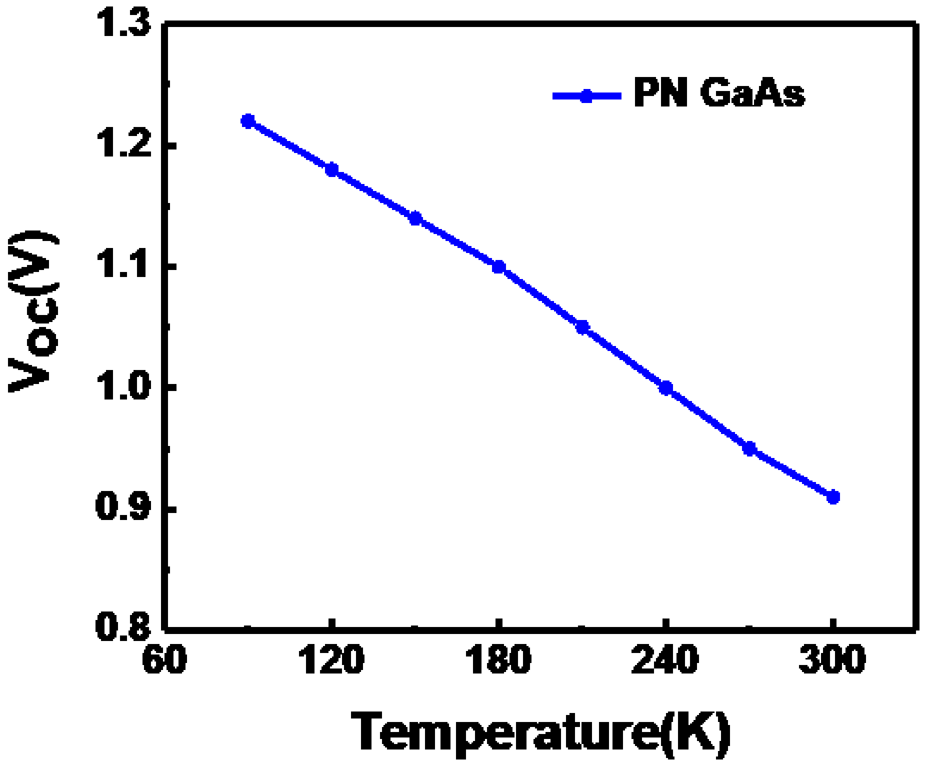

Figure S3. V_{oc} vary with different temperature based on the PN GaAs solar cell.

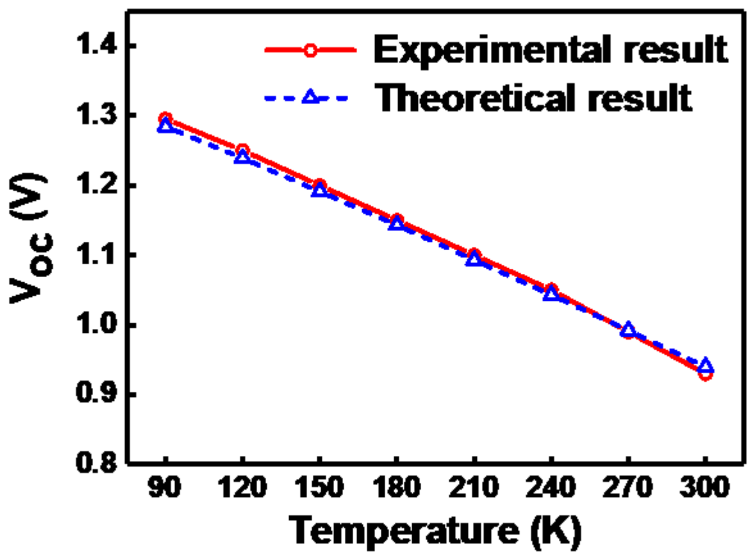

Figure S4. Comparison between theoretical calculation results and experimental results of PN GaAs solar cell under the various temperature.

Note S1: The calculation and discussion of the depletion region of graphene/GaAs and graphene/QWs/GaAs.

The width of depletion region of graphene/GaAs van der Waals heterojunction could be calculated as follows:

$$W = \left[\frac{2\varepsilon_0 \varepsilon_r V_{bi}}{qN_d}\right]^{\frac{1}{2}} \quad (1)$$

$$V_{bi} = \frac{W_s - W_m}{q} \quad (2)$$

$$W_s = \chi + \frac{kT}{q} ln \frac{N_c}{N_d} \quad (3)$$

where W is the width of depletion region, ε_0 is vacuum permittivity, ε_r is relative permittivity of semiconductor, V_{bi} is the build-in potential difference, q is the elementary charge, N_d is carrier concentration, W_s is the work function of semiconductor, W_m is the work function of metal, χ is electronic affinity, k is Boltzmann constant, T is absolute temperature, q is the electron charge, N_c is the effective density of states in conduction band. According to the equation (1)-(3), the width of depletion region of graphene/GaAs heterostructure is about 90.2 nm. The total thickness of periodic quantum wells combined with 5 nm $GaAs_{0.75}P_{0.25}$ and 3 nm $In_{0.15}Ga_{0.85}As$ is 80 nm. Additionally, the quantum wells are all intrinsic semiconductor with little doped. When graphene is assembled with GaAs grown with periodic quantum wells, the depletion region will fall almost in the intrinsic region, where the build-in potential difference is decided by the graphene and GaAs. So, the strength of built in

electric field is almost unchanged by integrating 80 nm periodic quantum wells.

Note S2: The calculation and discussion of conventional PN GaAs solar cell

The low temperature experiments based on the conventional bulk PN GaAs solar cell have also been designed. For PN GaAs solar cell, the band gap E_g can be expressed as the followed equation:

$$E_g(T) = E_g(0) - \frac{\alpha T^2}{T+\beta} \qquad (4)$$

$E_g(T)$ and $E_g(T)$ are the energy gap at temperature *T* and 0 K, respectively. The constant α and β depend on the type of material. As for GaAs, the parameter $E_g(0)$ = 1.519 eV, α = 5.405×10^{-4} eV/K, β = 204 K. The relationship between V_{oc}, J_{sc}, E_g and T can be descripted by:

$$\frac{\partial V_{oc}}{\partial T} = \frac{1}{T}\left[V_{oc} - \frac{E_g}{q} - \frac{3kT}{q}\right] + \frac{1}{q}\cdot\frac{\partial E_g}{\partial T} + \frac{kT}{qJ_{sc}}\cdot\frac{\partial J_{sc}}{\partial T} \qquad (5)$$

According to the experimental result (Figure S3, Supporting Information), $\frac{\partial J_{sc}}{\partial T} \approx 0$. So, the V_{oc} mainly changes with the width of the band gap. Further to calculate and simplify, the theoretical results and the experimental results are well matched, which has been showed in Figure S4.
